# Food Insecurity and Cardiovascular Health in Pregnant Women: Results From the Food for Families Program, Chelsea, Massachusetts, 2013–2015

**DOI:** 10.5888/pcd13.160212

**Published:** 2016-11-03

**Authors:** Mary E. Morales, Michael H. Epstein, Danelle E. Marable, Sarah A. Oo, Seth A. Berkowitz

**Affiliations:** Author Affiliations: Mary E. Morales, Harvard Medical School, Division of General Internal Medicine, Massachusetts General Hospital, Boston, Massachusetts; Michael H. Epstein, Danelle E. Marable, Center for Community Health Improvement, Massachusetts General Hospital, Boston, Massachusetts; Sarah A. Oo, Center for Community Health Improvement, Massachusetts General Hospital, Massachusetts General Hospital Chelsea HealthCare Center, Chelsea, Massachusetts. Dr Berkowitz is also affiliated with Harvard Medical School, the Division of General Internal Medicine and the Diabetes Center, Massachusetts General Hospital, Boston, Massachusetts.

## Abstract

**Background:**

Food insecurity, uncertainty about the ability to acquire adequate food, is associated with cardiometabolic disease in pregnant women. Whether food insecurity interventions improve cardiometabolic health is unknown.

**Methods:**

We conducted a retrospective analysis of women who visited the obstetrics clinic in a community health center from 2013 through 2015. Patients could be referred to the Food for Families (Food for Families) program, which connects food insecure women to food resources. We hypothesized that participation in Food for Families would be associated with better blood pressure and blood glucose trends during pregnancy. We used a propensity score–matched design to reduce bias from differential entry into Food for Families.

**Results:**

Eleven percent of women who visited the obstetrics clinic were referred to Food for Families. In propensity score–matched analyses, we found no difference in baseline systolic blood pressure (SBP) between those who were referred and enrolled in Food for Families (113.5 mm Hg), those who were referred and did not enroll in Food for Families (113.9 mm Hg), and those who were not referred to Food for Families (114 mm Hg) (*P* = .79). However, during pregnancy, women who were referred to and enrolled in Food for Families had a better SBP trend (0.2015 mm Hg/wk lower, *P* = .006). SBP trends did not differ between women who were referred and did not enroll in Food for Families and those who were not referred. We observed no differences in blood glucose trends between groups (*P* = .40).

**Conclusions:**

Food for Families participation was associated with better blood pressure trends in pregnant women but no differences in blood glucose trends. Food insecurity reduction programs may improve cardiovascular health for vulnerable pregnant women, and this topic deserves further study incorporating randomized program entry.

## Introduction

Food insecurity, as defined by the United States Department of Agriculture (USDA), represents limited or uncertain access to adequate food (1). Overall, 14% of US households are food insecure, but food insecurity is concentrated in households with children (19% food insecure), households headed by non-Hispanics blacks (26%) and Hispanics (22%), and low-income households (33%) (1).

Previous research showed that food insecurity is associated with both the presence and suboptimal control of cardiometabolic diseases, such as hypertension and diabetes (2–10), which are leading causes of illness and death in the United States (11). Food insecurity has health implications during pregnancy. High blood pressure in pregnancy is associated with poor outcomes for both the mother and child (12). Furthermore, food insecurity is associated with gestational diabetes (13) and poor birth outcomes, including low birthweight and increased risk of birth defects such as cleft palate and spina bifida (14,15). However, it is not known whether programs to combat food insecurity improve cardiovascular health in pregnant women. Therefore, we sought to determine whether participation in a food insecurity reduction program, the Food for Families program, improved blood pressure and blood glucose levels among pregnant women. We hypothesized that participation would be associated with better blood pressure and blood glucose levels in pregnant women than in those who did not participate.

## Methods

### Setting and study sample

All study participants received obstetrical care at a community health center in Chelsea, Massachusetts, that is affiliated with an academic medical center. Chelsea is a diverse, low-income community: 24% of the population lives below the federal poverty level, 44% were born outside the United States, and 68% speak a language other than English at home (16). However, health insurance coverage is high (16), and all low-income pregnant women in Massachusetts qualify for health insurance, regardless of immigration status (17).

For this retrospective study, all pregnant adult women, aged 18 years or older, who visited the obstetric clinic at the Chelsea Healthcare Center from June 1, 2013, through June 1, 2015, were included. Study data were extracted from the electronic health record by using previously validated algorithms (18–20). This study was approved by the institutional review board at Partners Healthcare.

Food for Families is an interventional program that identifies food insecure patients and connects them with food resources, such as the Supplemental Nutrition Assistance Program (SNAP), the Special Supplemental Nutrition Program for Women, Infants, and Children (WIC), and food pantries. Participants were identified in 2 ways — screening using a standardized assessment form at visit check-in or by referral from a provider if food insecurity was uncovered during the course of a visit (21). Once patients were referred to Food for Families, those who choose to enroll completed a standardized enrollment interview. Patients were then assisted with obtaining food resources tailored to their specific situation, considering patient preferences, cultural appropriateness, where patients lived, and program eligibility. Examples of assistance were support with SNAP or WIC enrollment or provision of information regarding local food pantries.

Because it is strongly associated with poor health outcomes, both during pregnancy and after, the primary outcome for this study was blood pressure trend during pregnancy (12). The secondary outcome was trend in blood glucose. Data on systolic blood pressure (SBP), diastolic blood pressure (DBP), and blood glucose level was extracted from the electronic medical record.

We extracted data on several factors related to food insecurity and cardiovascular health, such as race/ethnicity, language, and marital status. For patients who completed a Food for Families enrollment interview, we also extracted data on household size, housing status, annual income, SNAP eligibility, WIC participation, use of food pantries, use of free meal programs or soup kitchens, health insurance, self-reported health status, and whether patients wanted to make changes to the way they ate and whether they wanted more information on eating healthy food on a budget. Income-to-poverty ratio was calculated as reported income, in dollars, divided by poverty threshold, in dollars, for household size of respondent in year of interview. Poverty thresholds can be found at the US Census website (http://www.census.gov/data/tables/time-series/demo/income-poverty/historical-poverty-thresholds.html).

Because neighborhood factors such as food access, poverty, and unemployment may also affect health, we geocoded patient addresses at the census tract level and linked this to sociodemographic and food access variables available from the US Census Bureau (http://www.factfinder.census.gov) by using the 2009–2013 American Community Survey 5-year estimates, and the USDA Food Research Atlas (http://www.ers.usda.gov/data-products/food-access-research-atlas).

### Statistical analysis

We first conducted descriptive statistics and compared groups by using χ^2^ tests for categorical variables and *t* tests for continuous variables. We next created intensity maps to explore potential overlap between the distribution of food insecurity and neighborhood factors using US Census and USDA data. We used Fusion Tables (https://www.google.com/fusiontables) for data visualization.

Because the Food for Families program is designed to allow clinicians to channel more severely food insecure patients into it, we were concerned that this could introduce bias when comparing those referred to Food for Families versus those who were not. To help mitigate this bias, we used a propensity score matching approach. The propensity score predicted the likelihood of Food for Families program referral using sociodemographic, clinical, and neighborhood data. We then matched participants who were and were not referred to Food for Families in a 1:1 ratio using a “greedy” matching algorithm (22). We used this matched cohort to compare trends in SBP, DBP, and blood glucose level for those referred to Food for Families who enrolled, compared with those who were referred but did not enroll, and those who were not referred. As a negative control, we also compared trends in those who were referred, regardless of enrollment status, with those who were not, hypothesizing that those who were referred but did not enroll in the program should receive no benefit from treatment (ie, participation in the Food for Families program). For trend testing, we used longitudinal linear mixed-effects models, by using a patient-level random effects term to account for repeated measurements. To help account for potential confounding not addressed through propensity score matching, we also fit linear mixed-effects models adjusting for age, race/ethnicity, insurance, primary language, and census tract of residence (using fixed effects). All analyses were performed using SAS version 9.3 (SAS Institute, Inc).

## Results

A total of 1,295 pregnant women (aged ≥18 y) were seen in the obstetrics clinic during the study period. Among these women, 11% (145 of 1,295) were referred to Food for Families. Compared with patients not referred, those referred were more likely to be unmarried (*P* = .002), to be Hispanic (*P* < .001), and to speak Spanish (*P* < .001) (Table 1). In total, 67% (97 of 145) of referred women enrolled in Food for Families.

**Table 1:** Participant (N = 145) Demographics, by Referral Status, Food for Families Program, Chelsea, Massachusetts, 2013–2015

Characteristic	Referred to Food for Families, N = 145^a^	Not Referred to Food for Families, N = 1,150^a^	*P* Value^b^
**Age, y (standard deviation)**	30.1 (6.0)	30.3 (6.4)	.70
**Race/ethnicity**			
Non-Hispanic white	4.83	30.35	<.001
Non-Hispanic black	6.90	8.00	
Hispanic	84.83	55.48	
Asian/other/multiracial	3.45	6.17	
**Insurance**			
Private	47.59	66.70	.001
Medicare	1.38	0.96	
Medicaid	48.28	30.26	
Uninsured	2.76	2.09	
**Language**			
English	14.48	54.70	<.001
Spanish	77.24	34.43	
Other	8.28	10.87	
**Marital status**			
Single	62.76	44.61	.002
Married/partnered	33.79	51.30	
Legally separated/divorced/widowed	3.45	4.09	

a Values are percentages unless otherwise indicated.

b *P* values were calculated by using χ^2^ tests for categorical variables and *t* tests for continuous variables.

Among Food for Families participants, 71% had annual incomes below the federal poverty level and 46% reported housing insecurity as indicated by renting a room or living with relatives or friends (Table 2). Forty-nine percent had Medicaid insurance, 43% were eligible for SNAP, and 87% were enrolled in WIC. The majority had never made use of a free meal program or soup kitchen (78%) or a food pantry (75%). When asked to self-report their health status, 69% rated their current health as good, very good, or excellent. Thirty-five percent expressed a desire to learn more about eating healthy food on a budget.

**Table 2:** Sociodemographic Characteristics of Participants (N = 97), Food for Families Program, Chelsea, Massachusetts, 2013–2015

Characteristic	% of Participants
**Income-to-poverty ratio^a^**	
<0.5	39
0.5–1	32
1–1.5	11
1.5–2	4
>2	2
Information missing	11
**Type of housing**	
House — own	3
Apartment — rent	49
Room rental	36
Live with relatives or friends	10
Information missing	1
**Health insurance**	
MassHealth (Medicaid)	49
Neighborhood health plan	21
Free care	3
Other	9
Information missing	18
**SNAP eligible**	
Yes	43
No	29
Information missing	28
**WIC participation**	
Yes	87
No	3
Information missing	10
**Has ever used a food pantry**	
Yes	5
No	75
Information missing	20
**Has ever used a free meal program or soup kitchen**	
Yes	0
No	78
Information missing	22
**Self-reported health status**	
Excellent	4
Very good	26
Good	39
Fair	9
Poor	4
Information missing	18
**Would like more information on eating healthy food on a budget **	
Yes	35
No	13
Information missing	52

Abbreviations: SNAP, Supplemental Nutrition Assistance Program; WIC, Special Supplemental Nutrition Assistance Program for Women, Infants, and Children.

a Income-to-poverty ratio was calculated as reported income, in dollars, divided by poverty threshold, in dollars, for household size of respondent in year of interview. Poverty thresholds can be found at the US Census website (www.census.gov/data/tables/time-series/demo/income-poverty/historical-poverty-thresholds.html).

**Figure 1:**
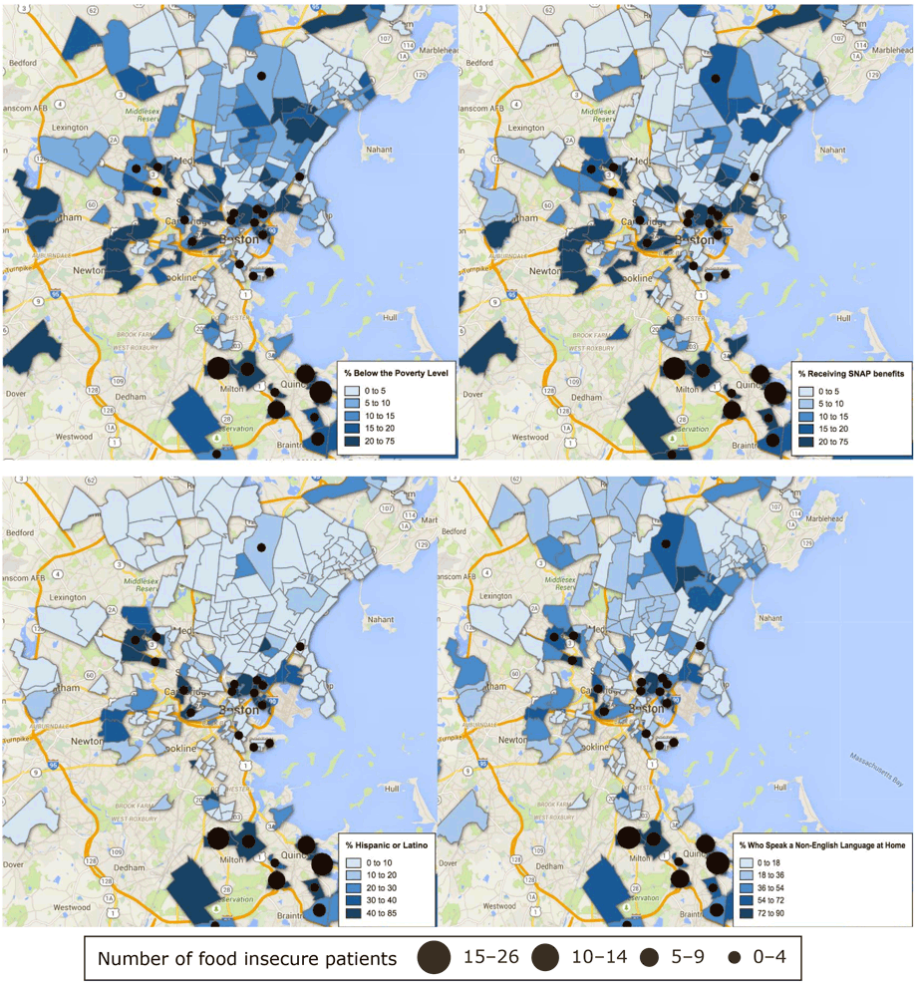
Intensity maps of sociodemographic variables at the census tract level with overlapping data on the number of food insecure women residing in each census tract, Boston metropolitan area, 2013–2015. Abbreviation: SNAP, Supplemental Nutrition Assistance Program.

Figure 1 shows intensity maps for sociodemographic variables by census tract, with overlapping data on the number of food insecure women residing in each census tract. The majority of women seen in the obstetric clinic at the Chelsea Health Clinic resided in 5 census tracts. These 5 census tracts also had the highest number of food insecure patients. The census tracts with the highest number of food insecure patients had high prevalence of people living below the federal poverty level, SNAP participation, Hispanics, and people who speak a non-English language at home.

**Figure 2:**
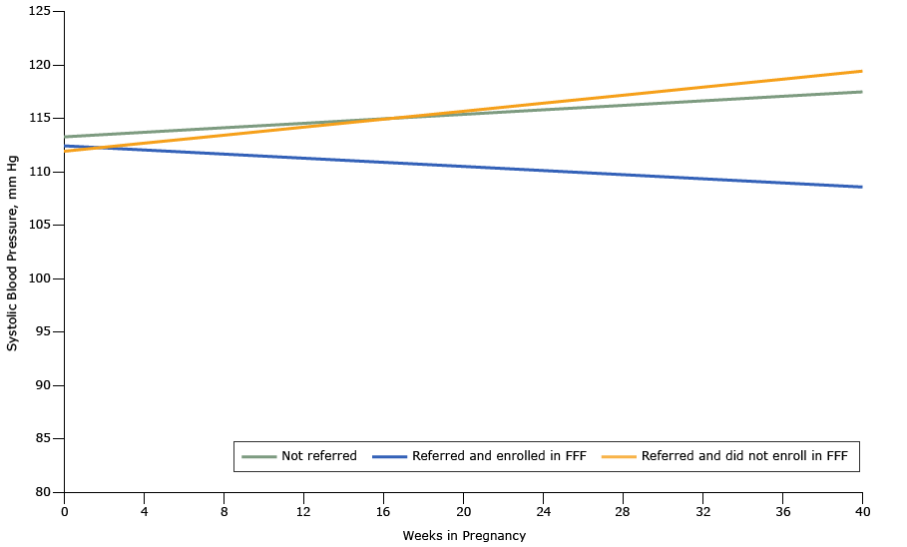
Systolic blood pressure trends among women in the obstetric clinic of the Chelsea Health Clinic during the course of pregnancy based on propensity score–matched analyses, Chelsea, Massachusetts, 2013–2015. Abbreviation: FFF, Food For Families.

**Figure 3:**
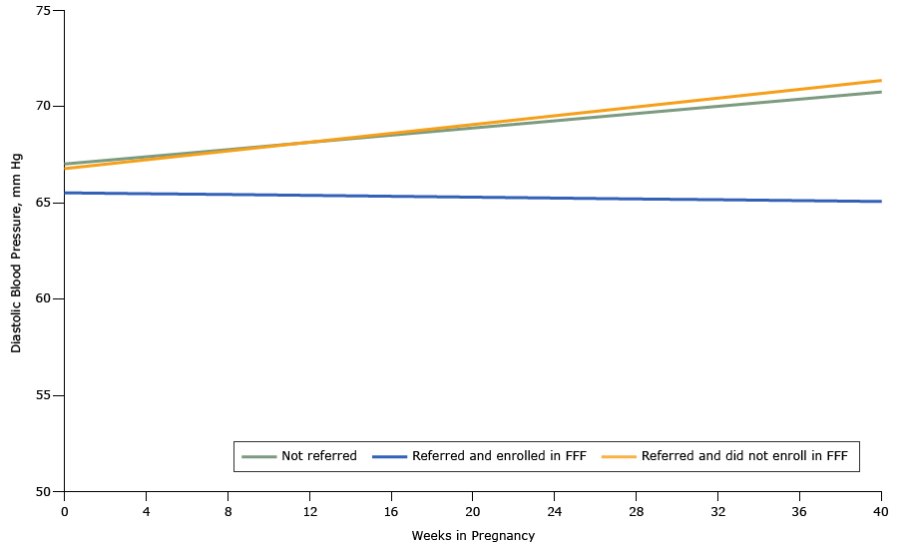
Diastolic blood pressure trends among women in the obstetric clinic of the Chelsea Health Clinic over the course of pregnancy based on propensity score-matched analyses, Chelsea, Massachusetts, 2013–2015. Abbreviation: FFF, Food For Families.

**Figure 4:**
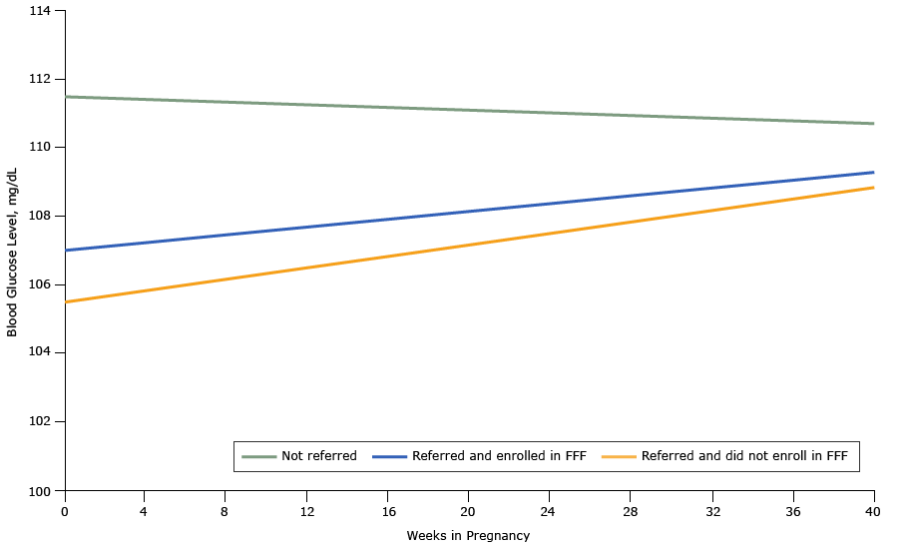
Blood glucose trends among women in the obstetric clinic of the Chelsea Health Clinic during the course of pregnancy based on propensity score–matched analyses, Chelsea, Massachusetts, 2013–2015. Abbreviation: FFF, Food For Families.

At baseline in the propensity score–matched cohort, SBP, DBP, and blood glucose levels were similar comparing those who were referred and enrolled in Food for Families, those who were referred but did not enroll in Food for Families, and those who were not referred to Food for Families (Table 3). When comparing those referred to Food for Families with a propensity score–matched cohort who were not, regardless of enrollment status, we found no difference in blood pressure trend during the pregnancy (Figures 2 and 3) and (Table 4). However, during the course of their pregnancy, women who were referred to and enrolled in Food for Families had a better SBP (0.2015 mm Hg/wk lower, *P* = .006) and DBP (0.1049 mm Hg/wk lower, *P* = .02) trend than those who were not referred. Women who were referred to Food for Families but did not enroll did not show a difference in SBP or DBP trend compared with those who were not referred. We did not observe differences in blood glucose trend, either at baseline or over the course of the pregnancy, among groups (Figure 4). All results in models adjusted for age, race/ethnicity, insurance, primary language, and census tract of residence were similar to unadjusted results (Table 5). 

**Table 3:** Propensity Score–Matched Analyses of Systolic Blood Pressure, Diastolic Blood Pressure, and Blood Glucose for Participants (N = 290), at Baseline, by Referral, and by Enrollment, Food for Families Program, Chelsea, Massachusetts, 2013–2015

Variable	Participant Status, by Referral Status				
	Referred to Food for Families (n = 145)		Not Referred to Food for Families (n = 145)		*P* Value^a^
	Baseline, mean				
Systolic blood pressure, mm Hg	113.7		114.0		.84
Diastolic blood Pressure, mm Hg	66.7		66.8		.94
Blood glucose, mg/dL	106.8		108.3		.68
**Variable **	**Participant Status, by Enrollment Status**				
	**Referred and Enrolled in Food for Families**		**Referred and Did Not Enroll in Food for Families**		**Not Referred to Food for Families,** **Mean**
	**Baseline, Mean**	***P* Value^b^**	**Baseline, Mean**	***P *Value^b^**	

Systolic blood pressure, mm Hg	113.5	.79	113.9	.99	114
Diastolic blood pressure, mm Hg	66.2	.74	67.9	.49	66.7
Blood glucose, mg/dL	107.9	.93	104.3	.47	108.3

a *P* values are from linear mixed models.

b Compared with control group (women who were not referred to Food for Families).

**Table 4:** Trends Over Time in Propensity Score–Matched Analyses of Systolic Blood Pressure (SBP), Diastolic Blood Pressure (DBP), and Blood Glucose of Participants (N = 290), by Referral and Enrollment Status, Food for Families Program, Chelsea, Massachusetts, 2013–2015

Variable	Participant Status, by Referral Status		*P* Value^a^
	Trend	Difference in Trend	
**Systolic blood pressure, mm Hg/wk **			
Referred to Food for Families	0.01447	−0.09073	.08
Not referred to Food for Families	0.1052	NA	NA
**Diastolic blood pressure, mm Hg/wk **			
Referred to Food for Families	0.03966	−0.05409	.17
Not referred to Food for Families	0.09375	NA	NA
**Blood glucose, mg/dL/wk**			
Referred to Food for Families	0.02303	−0.03392	.66
Not referred to Food for Families	0.05695	NA	NA
**Variable**	**Participant Status, by Enrollment Status**		
	**Trend**	**Difference in Trend^b^**	***P* Value^c^**
**Systolic blood pressure, mm Hg/wk**			
Food insecure and enrolled in Food for Families	−0.0964	−0.2015	.006
Food insecure and did not enroll in Food for Families	0.18715	0.08205	.24
Not referred to Food for Families	0.1051	NA	NA
**Diastolic blood pressure, mm Hg/wk **			
Food insecure and enrolled in Food for Families	−0.01131	−0.1049	.02
Food insecure and did not enroll in Food for Families	0.11442	0.02083	.70
Not referred to Food for Families	0.09359	NA	NA
**Blood glucose, mg/dL/wk **			
Food insecure and enrolled in Food for Families	−0.01954	−0.07646	.40
Food insecure and did not enroll in Food for Families	0.08363	0.02671	.78
Not referred to Food for Families	0.05692	−NA	NA

Abbreviation: NA, not applicable.

a *P* values calculated by using linear mixed models.

b Compared with not referred to Food for Families.

c For difference in trend, compared with not referred to Food for Families.

**Table 5:** Propensity Score-Matched Analyses of Systolic Blood Pressure, Diastolic Blood Pressure, and Blood Glucose of Participants (N = 290), by Referral and Enrollment Status, Adjusted for Race/Ethnicity, Insurance, Primary Language, and Census Tract of Residence, Food for Families Program, Chelsea, Massachusetts, 2013–2015^a^^,^ b

Variable	Trend	Difference in Trend	*P* Value
**By Referral Status**			
**Systolic blood pressure, trends over time, mm Hg/wk**			
Referred to Food for Families	0.01846	−0.07625	.14
Not referred to Food for Families	0.09471	—	—
**Diastolic blood pressure, trends over time, mm Hg/wk**			
Referred to Food for Families	0.03882	−0.03867	.33
Not referred to Food for Families	0.07749	—	—
**Blood glucose, trends over time, mg/dL/wk**			
Referred to Food for Families	−0.00279	−0.00929	.91
Not referred to Food for Families	0.006505	—	—
**By Enrollment Status**			
**Systolic blood pressure, trends over time, mm Hg/wk^a^**			
Food insecure and enrolled in Food for Families	−0.09979	−0.1946	.001
Food insecure and did not enroll in Food for Families	0.20301	0.1082	.12
Not referred to Food for Families	0.09481	—	—
**Diastolic blood pressure, trends over time, mm Hg/wk**			
Food insecure and enrolled in Food for Families	−0.02402	−0.1013	.03
Food insecure and did not Enroll in Food for Families	0.13436	0.05708	.29
Not Referred to Food for Families	0.07728	—	—
**Blood glucose, trends over time, mg/dL/wk**			
Food insecure and enrolled in Food for Families	−0.03032	−0.03597	.71
Food insecure and did not enroll in Food for Families	0.050794	0.04514	.65
Not referred to Food for Families	0.005654	—	—

Abbreviation: —, not applicable.

a Results adjusted for age, race/ethnicity, insurance, primary language, and census tract of residence.

b Difference in trend and *P* values is for difference compared with women not referred to Food for Families.

## Discussion

We found that participation in Food for Families was associated with modestly better blood pressure trends during pregnancy. Propensity score–matched patients who were not referred to Food for Families and those who were referred but did not enroll experienced a rise in blood pressure during pregnancy, whereas those who enrolled in Food for Families did not. We observed no difference in blood glucose levels between groups.

The results of this study are consistent with, and extend, those of previous studies on food insecurity and cardiovascular health. Previous reports have found that food insecurity is associated with the cardiovascular risk factors of hypertension, diabetes, elevated cholesterol, and obesity (6,23,24). A previous study of a hypertension intervention that did not address food insecurity found that those experiencing food insecurity did not benefit from the intervention, whereas those who were food secure did (7). Previous studies in pregnant women have shown that food insecurity is associated with maternal cardiovascular risk factors and poor birth outcomes (14,15). In a sample of 810 pregnant women, Laraia et al found that food insecurity is associated with prepregnancy obesity, higher gestational weight gain, and gestational diabetes, but not pregnancy-induced hypertension (13). A study of 526 women found that food insecurity was associated with prepregnancy weight status, and among women who were overweight or obese prepregnancy, food insecurity was associated with greater weight gain and a higher body mass index (weight in kg/height in m^2^) at 12 months postpartum (25). These risks warrant studies to determine whether food insecurity-reduction programs can improve cardiovascular health.

This study has several public health and clinical implications. The results of this study suggest that food insecurity–reduction programs can improve cardiovascular health in pregnant women. In particular, WIC participation was high among those who were referred to and enrolled in Food for Families. Nutritional assistance programs, such as WIC and SNAP, could be an important part of health maintenance for vulnerable pregnant women. If nutrition assistance programs do improve health, screening for food insecurity in obstetric care may be a useful tool to identify at-risk women. Ideally, this screening would be connected with efforts to assist patients in program enrollment. This also has implications for co-location of WIC offices with health care providers for vulnerable women.

The results of this study should be interpreted in the context of several limitations. First, entry into the Food for Families program was nonrandom. Although we attempted to account for confounding introduced by this with the use of a propensity score–matching approach, residual confounding affecting who was referred and enrolled in the program may be present. Additionally, because food insecurity was only assessed at baseline, we do not know whether results attributable to the program were due to a reduction in food insecurity or some other factor. At the least, however, these results would justify a randomized interventional study to further evaluate the efficacy and mechanisms of this approach. Second, there were limitations in the data set. This study relied on data collected in routine care, which can introduce variability into measurements of blood pressure and blood glucose. However, these data are what clinicians rely on to provide care. Furthermore, we did not have access to pharmacy records, which limited our understanding of medications participants may have been taking and how that may have affected the results we observed. Additionally, we had access to detailed sociodemographic data only for participants who enrolled in Food for Families. Although we observed high WIC participation among enrollees, we do not know whether this was differentially higher than in those who did not enroll and therefore may have mediated the observed association. Third, this study included data from a single health care system, which may limit generalizability. Also, available sample sizes were too small to study rare outcomes, such as cardiovascular events, or changes in birth outcomes that may be attributable to improved cardiovascular health. Finally, mean blood glucose levels were low, and there were few cases of gestational diabetes in any group, which may have reduced the power to detect differences in blood glucose trends.

The limitations of this study were balanced by several strengths. This study focused on pregnant women, who are understudied with regard to the relationship between health and food insecurity. Furthermore, this study included high numbers of racial/ethnic minority and low-income participants, who are also understudied. We also accounted for a range of both individual and neighborhood-level factors that could influence health outcomes. Finally, lack of observed benefit in those who did not participate strengthens our confidence that program participation led to better blood pressure levels, and though one may argue that those who enrolled in Food for Families were likely to be more engaged in their care than those who did not, the fact that we did not observe a difference in blood glucose trends strengthens the findings with regard to blood pressure.

In this study we found that referral to and enrollment in the Food for Families program was associated with better trends in blood pressure during pregnancy. This finding suggests that food insecurity–reduction programs may improve the cardiovascular health of pregnant women. Future studies should include randomization, address which program elements are effective at promoting cardiovascular health, and assess whether program modifications or additions could also improve blood glucose levels or other aspects of cardiovascular health. Programs that address food insecurity in pregnant women may play an important role in improving health in vulnerable populations.
