# Erratum, Vol. 13, November 3, 2016

**DOI:** 10.5888/pcd13.160212e

**Published:** 2016-11-23

In the article “Food Insecurity and Cardiovascular Health in Pregnant Women: Results From the Food for Families Program, Chelsea, Massachusetts, 2013–2015,” Figure 4 was inadvertently mislabeled and was replaced with a corrected figure on November 9, 2016. The corrected article appears at http://www.cdc.gov/pcd/issues/2016/16_0212.htm. We apologize for any confusion this error may have caused.
